# Relationship between postural control and restricted, repetitive behaviors in autism spectrum disorders

**DOI:** 10.3389/fnint.2013.00028

**Published:** 2013-05-07

**Authors:** K. J. Radonovich, K. A. Fournier, C. J. Hass

**Affiliations:** ^1^Division of General Pediatrics, Department of Pediatrics, University of FloridaGainesville, FL, USA; ^2^Department of Kinesiology, University of Rhode IslandKingston, RI, USA; ^3^Applied Neuromechanics Laboratory, Applied Physiology and Kinesiology, University of FloridaGainesville, FL, USA

**Keywords:** autism spectrum disorders (ASD), center of pressure (COP), repetitive behavior, posture, stability

## Abstract

Restricted, repetitive behaviors (RRBs) are one of the core diagnostic criteria of autism spectrum disorders (ASD), and include simple repetitive motor behaviors and more complex cognitive behaviors, such as compulsions and restricted interests. In addition to the core symptoms, impaired movement is often observed in ASD. Research suggests that the postural system in individuals with ASD is immature and may never reach adult levels. RRBs have been related to postural sway in individuals with mental retardation. Our goals were to determine whether subjects with ASD had greater postural sway and whether RBS-R scores were related to the magnitude of postural sway. We compared the center of pressure (COP) sway area during quiet stance with scores on the Repetitive Behavior Scale-Revised (RBS-R) in children with ASD and typically developing (TD) controls ages 3–16. All subjects had Non-verbal IQ > 70. Subjects performed four quiet stance trials at a self-selected stance width for 20 s. Subjects with ASD had greater postural sway area compared to controls. Not surprisingly, subjects with ASD exhibited greater frequencies and intensities of RRBs overall and on all six subscales. Further, there was a positive correlation between postural sway area and presence of RRBs. Interestingly, results of the postural sway area for the ASD group suggests that roughly half of the ASD subjects scored comparable to TD controls, whereas the other half scored >2 SD worse. Motor impaired children did not have significantly worse IQ scores, but were younger and had more RRBs. Results support previous findings of relationships between RRBs and postural control. It appears that motor control impairments may characterize a subset of individuals with ASD. Better delineation of motor control abilities in individuals with ASD will be important to help explain variations of abilities in ASD, inform treatment, and guide examination of underlying neural involvement in this very diverse disorder.

## Introduction

Restricted interests and repetitive, stereotyped behaviors (RRBs) are one of the three core diagnostic areas of autism spectrum disorders (ASD), along with impairments in communication and social interaction (APA, 2000). The restricted interests and repetitive behaviors seen in individuals with ASD include a broad class of behaviors that are characterized by their repetitiveness and invariance, including simple repetitive motor behaviors (e.g., hand flapping, rocking/swaying, spinning) and restricted interests, (e.g., specific object attachments, compulsions, rituals, and routines, an “anxiously obsessive desire for sameness”) (Kanner, 1943). Research supports the conceptualization of two distinct types of repetitive behaviors: “lower order” sensory and motor repetitive behaviors and “higher-order” behaviors marked by cognitive rigidity (Turner, 1999). A factor analysis of RRBs by Lam et al. (2008) replicated these two factors, but also found a third factor characterized by circumscribed interests.

In addition to the three core symptoms of ASD, impaired movement is commonly observed in individuals with ASD. In fact, motor control impairments are the most frequently reported non-verbal findings in ASD (Noterdaeme et al., 2002). Individuals with ASD have been described as having greater clumsiness and motor coordination abnormalities (Vilensky et al., 1981; Jones and Prior, 1985; Rapin, 1997; Ghaziuddin and Butler, 1998), although findings have been inconsistent. Several studies have failed to find motor differences between children with ASD and those with learning disabilities or mental retardation (Morin and Reid, 1985), general developmental delay (Provost et al., 2007), and language disorders (Noterdaeme et al., 2002). Other studies of movement in ASD have revealed impairments in a wide variety of abilities, including balance, gait, manual dexterity, ball skills, and object control (Vilensky et al., 1981; Jones and Prior, 1985; Bauman, 1992; Kohen-Raz et al., 1992; Hallett et al., 1993; Rogers et al., 1996; Rapin, 1997; Ghaziuddin and Butler, 1998; Molloy et al., 2003). For example, children with ASD have been shown to have reduced stride lengths and increased stance times during gait (Vilensky et al., 1981). Examination of motor abilities associated with subtle neurological signs determined that boys with ASD had worse balance and gait, slower speed and more dysrhythmia with timed movements of the hands and feet, and presence of more overflow movements during speeded limb movements and stressed gait maneuvers than age-matched peers (Jansiewicz et al., 2006). Others have purported impairments in the planning and execution of movement in children with ASD (Glazebrook et al., 2006; Rinehart et al., 2006). Motor control problems on standardized assessments in children with ASD have been reported in children as young as 20 months of age (Provost et al., 2007). Retrospective videotape analysis of motor development suggests that abnormal motor abilities, such as abnormal righting and rolling over, may be evident in infancy for children who are later diagnosed with ASD (Teitelbaum et al., 1998; Baranek, 1999). In summary, motor findings in ASD seem to appear very early in life and are present across a wide variety of tasks and abilities. Commonly referred to as “clumsiness,” it is unclear whether these deficits are specific to autism, and, if so, how these observed motor impairments are related to the core diagnostic symptoms of autism.

In order to begin to more objectively describe the reported “clumsiness” in autism, we chose to examine postural stability in children with ASD because numerous studies have identified deficits in postural control in ASD. Assessments of postural stability, whereby sensory input was modulated, have particularly demonstrated decreased postural stability in individuals with ASD as compared with controls (Kohen-Raz et al., 1992; Gepner et al., 1995; Molloy et al., 2003; Minshew et al., 2004). However, our group found that even when sensory inputs are not modified, postural stability during quiet stance has been shown to be impaired in children with ASD (Fournier et al., 2010). While Minshew et al. (2004) found reduced postural stability for quiet stance, they also found that postural stability was particularly reduced in conditions in which somatosensory input was disrupted, by moving the support and/or changing visual input. Overall, research suggests that the postural system in individuals with ASD is immature and may never reach adult levels (Kohen-Raz et al., 1992; Minshew et al., 2004). Taken together, results of postural instability in ASD are consistent with a deficit in the integration of visual, vestibular, and somatosensory input to maintain postural orientation (Molloy et al., 2003; Minshew et al., 2004).

An immature postural system can be a limiting factor on the execution of other motor skills. For example, data from a bimanual lift task suggested that children with ASD rely on reactive postural control when performing lifting tasks, rather than on the typical anticipatory postural control used in typical controls (Schmitz et al., 2003). Fournier et al. (2010) also showed that dynamic postural stability was impaired, such that children with ASD made significantly smaller lateral center of pressure (COP) shifts when initiating gait. Interestingly, there were no differences found in the posterior-anterior COP shift, suggesting that the mechanism for generating forward momentum is intact in children with ASD in spite of impaired postural control.

Impaired stable posture and an immature postural control system during movement can be a limiting factor on the emergence of other motor skills (such as coordinated hand/head movements and inhibition of reflexes) and may constrain the ability to develop mobility and manipulatory skills (Shumway-Cook and Woollacott, 2001). Postural control requires a level of stability necessary prior to executing additional motor skills or activities. Thus, if children with ASD have impaired postural control, this could lead to difficulty with tasks involving fine motor control (e.g., writing, tying shoes), and social play (e.g., riding a bike, throwing a ball, and team sports) (Jansiewicz et al., 2006). Because postural stability is the basis for so many movements, further examination of postural instabilities in this population is needed to better explain observed motor impairments in ASD, and may be a first step toward determining the best approach for improving postural stability and related skills (mobility and manipulation).

Observations of impaired postural control and other motor skills lead us to consider how motor system involvement in ASD might be related to the core diagnostic criteria, in particular, the presence of repetitive motor behavior and restricted interests. Theories about repetitive motor behavior, also referred to as stereotypies, have largely focused on the presumed function or maintenance mechanisms of the behavior, such as reinforcement (Lovaas et al., 1987; Iwata et al., 1994), arousal modulation or anxiety reduction (Hutt and Hutt, 1965; Kinsbourne, 1980; Rodgers et al., 2012), homeostatic responding (Repp et al., 1992), and emotional regulation (Prizant et al., 2006; Janzen and Zenko, 2012).

A recent review of RRBs suggested that repetitive behavior likely occurs as the result of multiple etiologies or neurobiological factors (Lewis and Kim, 2009). The motor control theory of repetitive motor behavior suggests that, while the aforementioned functions may play a role in maintaining the engagement of repetitive motor behavior, they do not explain the origin of these movements (Bodfish et al., 2001). The motor control theory suggests that these repetitive behaviors occur as the result of a deficient motor system and its attempts to maintain homeostasis and engage in goal-oriented motor skills. In support of this, Bodfish et al. (2001) found that poor motor control, as measured by increased postural sway, was associated with increased motor stereotypies in individuals with mental retardation. As the Bodfish et al. (2001) study did not assess individuals with autism, we set out to determine if this relationship would be the same in individuals with ASD (and not mental retardation). Further, we evaluated whether postural sway would be correlated with more complex, cognitive, repetitive behaviors, in addition to motor stereotypies. In an effort to help further define the relationship between postural stability and RRBs, we compared postural sway and RRBs in children with ASD and typically developing (TD) children.

## Materials and methods

We assessed 18 children diagnosed with ASD (3.9–15.7 years) and 28 typically-developing (TD) control children (3.4–15.9 years) (see Table 1). Subjects with ASD were recruited from the University's Child and Adolescent Psychiatry Clinic and from the community. Clinical diagnoses of ASD (autistic disorder, Asperger disorder, or PDD, NOS) were initially determined by a licensed professional (psychologist or physician) and confirmed using the Autism Diagnostic Observation Schedule (ADOS; Lord et al., 1999) and the Social Communication Questionnaire (SCQ; Rutter et al., 2003). All subjects achieved scores of >70 on the Leiter-R Brief Non-verbal IQ (Roid and Miller, 1997). Children were excluded if known genetic/medical conditions, gross sensory deficits, use of assistive devices, or significant physical impairments were present. Furthermore, TD children were excluded if they had a history of a diagnosis of a psychiatric or neurological disorder. Participants in the TD group were equated to participants in the ASD group on chronological age, gender, and race. All subjects consented to the protocol, which was approved by an institutional review board, and children provided assent when appropriate.

**Table 1:** **Means and standard deviations (SD) for age, non-verbal IQ, and measures of COP variability during quiet stance**.

**Measure**	**ASD (*n* = 18)**		**TD (*n* = 28)**		***P*-value**
	**Mean**	***SD***	**Mean**	***SD***	
Age	8.18	3.4	8.31	4.0	0.905
Brief IQ	95.78	18.1	113.18	12.6	0.000^*^
COP_AREA_ (cm^2^)	27.59	35.7	6.01	6.66	0.003^*^

* *Significantly different at p < 0.05*.

Presence and severity of repetitive behaviors and restricted interests were assessed using the Repetitive Behavior Scale-Revised (RBS-R; Bodfish et al., 1999). The RBS-R is an empirical rating scale used to assess the presence and severity of repetitive behaviors (Stereotyped Behavior, Self-Injurious Behavior, Compulsive Behavior, Ritualistic Behavior, Sameness Behavior, and Restricted Behavior). The scale provides two separate scores for each of the six subscales and overall total. One score is an *intensity* score, a sum of the ratings for each item and the other score is a *frequency* score, a sum of the number of items endorsed or scored as present.

Postural control was assessed while participants stood quietly on a forceplate (Type 4060–10, Bertec Corp., Columbus, OH) embedded level to the floor. The laboratory was clutter-free, had a homogenous floor and was isolated from outside distractions with the use of monochromatic curtains. Subjects were instructed to stand as still as possible, with their arms at their side. Each participant performed four quiet stance trials at a self-selected stance width for 20 s. Foot positioning was marked on the initial trial and used for all subsequent trials. Ground reaction forces (GRF) and moments were recorded (360 Hz) from the forceplate. Trials where voluntary movements were observed were rejected and additional trials were performed. Trials were discounted if a participant engaged in a series of movements that indicated that they were no longer attending to the task of standing still (e.g., talking, picking up a foot, walking away, looking for their parent/guardian, reaching for a toy).

GRF and moments collected from the forceplate were used to calculate the instantaneous location of the COP. COP locations were then outputted for further analyses (Winter et al., 2003). Once outputted, the peak displacements of the COP in the mediolateral (ML Range) and anteroposterior (AP Range) directions were calculated. The sway area was determined by multiplying the peak displacements in the mediolateral and anteroposterior directions. Each subject's data from the four experimental trials were averaged to provide one representative score for each dependent variable.

Independent *t*-test analyses were conducted to identify differences between the groups for age and IQ. Due to the finding of a significant difference in IQ scores between the groups, further analyses used IQ as a covariate when identifying differences in the dependent variables (COP_AREA_, RBS-R scores) between children diagnosed with ASD and TD children. Correlational analyses were conducted on the RBS-R scores and postural sway area for the entire sample and then separately for each group. An *a' priori* alpha level of 0.05 was set for all statistical tests.

We had three primary questions of interest: (1) Is the magnitude of postural sway greater in children and adolescents with ASD compared to those TD? (2) Are RBS-R scores correlated with the magnitude of postural sway? and (3) Is this relationship more pronounced in ASD?

## Results

Results indicated that the two groups were similar in age [*t*_(1, 44)_ = 0.120, *p* > 0.05] and were of similar heights [*t*_(1, 44)_ = 0.193, *p* > 0.05]. However, the TD group had significantly higher non-verbal IQ scores [*t*_(1, 44)_ = 3.354, *p* < 0.05], thus IQ was used as a covariate in subsequent analyses (see Table 1).

**Figure 1:**
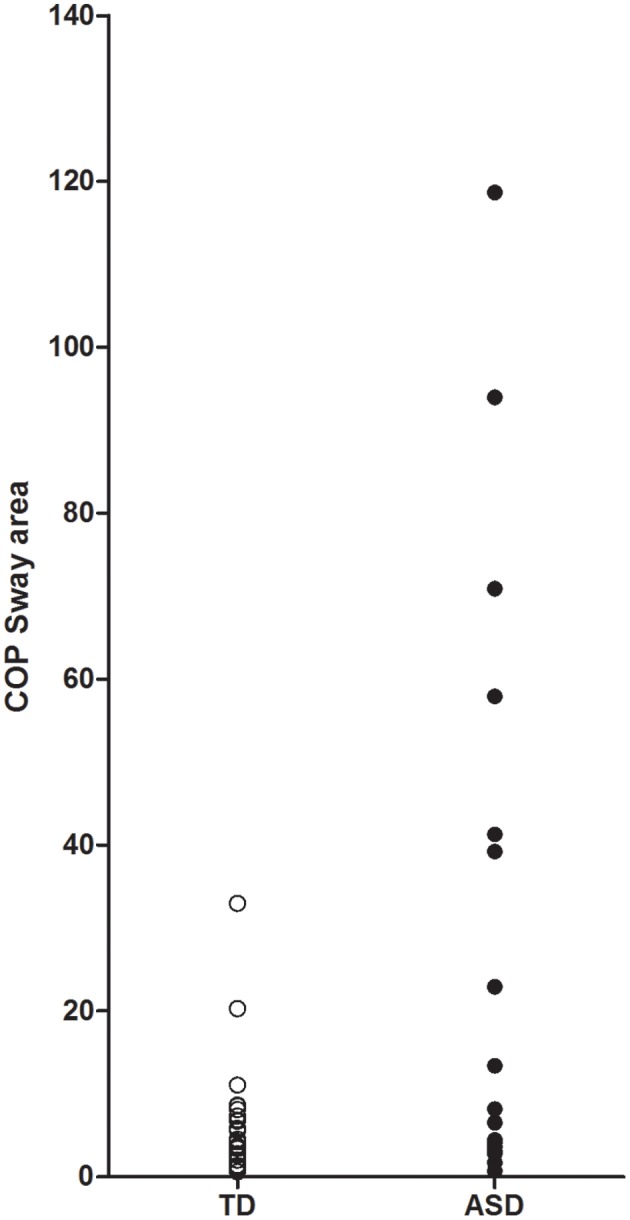
**Scatter plot of center of pressure (COP) sway area**.

Analysis of results on the postural sway area found that the distribution, particularly for the ASD group, was not normal (see Figure 1) and had a large positive skew. Therefore, we used bootstrapping in our analysis of covariance (ANCOVA) of postural sway. Bootstrapping uses a resampling procedure that uses random sampling with replacement to estimate distribution based on the population and is robust to violations of non-normality in the dependent variable. Results with ANCOVA, using IQ as a covariate, showed that the overall model considering group and IQ was significant, such that subjects with ASD had greater postural sway area compared to controls [*F*_(2, 43)_ = 6.738, *p* < 0.01] (see Figure 1). However, when considering the unique contribution of group [*F*_(1, 43)_ = 3.528, *p* > 0.05] or IQ [*F*_(1, 43)_ = 3.194, *p* > 0.05], neither independently significantly predicted sway. Of note, there was a trend toward significance for both group (*p* = 0.08) and IQ (*p* = 0.07).

As noted above, the distribution of postural sway area was not normal. When examining the individual postural sway data for the children with ASD (see Figure 1), it was noted that roughly half of children with ASD performed comparable to TD controls, whereas the other half performed >2 SD outside the TD range. We became interested in what might explain this large range of motor abilities in ASD. Therefore, we split the subjects into a group with “typical” sway and those with impaired sway (>2 SD). Preliminary analyses found that children with impaired sway had significantly worse IQ scores [*t*_(1, 44)_ = −2.914, *p* < 0.05] and were younger [*t*_(1, 44)_ = −2.101, *p* < 0.05] (see Table 2).

**Table 2:** **Means and standard deviations (SD) for Non-verbal IQ and age for groups based on postural stability**.

**Measure**	**Typical sway (*n* = 37)**		**Impaired sway (*n* = 9)**		***P*-value**
	**Mean**	***SD***	**Mean**	***SD***	
Age in years	9.14	1.3	6.01	2.5	0.041^*^
Brief IQ	95.78	18.1	113.18	12.6	0.006^*^

* *Significantly different at p < 0.05*.

For repetitive behaviors and restricted interests, not surprisingly, subjects with ASD exhibited greater frequencies and intensities of RRBs overall and on all six subscales (see Table 3). Children with ASD had increased frequency and intensity of RRBs over TD children at a range of 5 times to over 12 times greater.

**Table 3:** **Means and standard deviations (SD) for scores on RBS-R**.

**Scale**	**ASD (*n* = 18)**		**TD (*n* = 18)**		***P*-value**
	**Mean**	***SD***	**Mean**	***SD***	
**STEREOTYPED BEHAVIOR**					
Frequency	3.50	1.6	0.43	0.6	0.000^*^
Intensity	5.89	3.5	0.57	1.3	0.000^*^
**SELF-INJURIOUS BEHAVIOR**					
Frequency	1.89	2.3	0.14	0.4	0.000^*^
Intensity	2.61	3.2	0.14	0.4	0.000^*^
**COMPULSIVE BEHAVIOR**					
Frequency	3.56	2.1	0.68	1.5	0.000^*^
Intensity	5.89	4.3	1.07	2.9	0.000^*^
**RITUALISTIC BEHAVIOR**					
Frequency	3.89	1.7	0.68	1.2	0.000^*^
Intensity	7.06	3.9	0.96	2.3	0.000^*^
**SAMENESS BEHAVIOR**					
Frequency	6.00	3.1	0.64	1.3	0.000^*^
Intensity	10.22	7.9	0.82	2.0	0.000^*^
**RESTRICTED BEHAVIOR**					
Frequency	2.61	1.2	0.21	0.5	0.000^*^
Intensity	4.67	3.1	0.36	1.2	0.000^*^

* *Significantly different at p < 0.05*.

Overall, using Pearson correlation, our measure of postural control (sway area) was significantly correlated with the Total RBS-R frequency and intensity scores (*r* = 0.61, *p* < 0.01; *r* = 0.61, *p* < 0.01), as well as 5 out of the 6 subscale scores (*r* range of 0.46–0.62, all *p* < 0.01). Sway area was not related to the Self-injurious Behavior subscale (frequency *r* = 0.22, *p* > 0.05; intensity *r* = 0.13, *p* > 0.05).

Because the children in the TD group had such low rates of repetitive behaviors as assessed with the RBS-R we wondered if the correlation between postural sway and RRBs was different for children with ASD than for TD control. When examining the groups separately, these relationships did appear to be driven by the strong correlations within the group with ASD. For the ASD group, sway area was significantly correlated with the Total RBS-R frequency and intensity scores (*r* = 0.60, *p* < 0.01; *r* = 0.56, *p* < 0.05), as well as four out of the six subscale scores (all *p* < 0.05). In children with ASD, sway area was significantly correlated with the frequency and intensity of Stereotyped Behavior (*r* = 0.58, *p* < 0.05; *r* = 0.53, *p* < 0.05), Compulsive Behaviors (*r* = 0.67, *p* < 0.01; *r* = 0.69, *p* < 0.01), and Restricted Behavior (*r* = 0.60, *p* < 0.01; *r* = 0.67, *p* < 0.01), as well as the frequency of Sameness Behavior (*r* = 0.54, *p* < 0.05). Sway area for children with ASD was not related to the Self-injurious Behavior subscale (frequency *r* = −0.04, *p* > 0.05; intensity *r* = −0.15, *p* > 0.05) nor to the Ritualistic subscale (frequency *r* = 0.33, *p* > 0.05; intensity *r* = 0.45. *p* > 0.05). On the contrary, in controls, postural sway was only related to the frequency and intensity of Self-injurious Behavior (*r* = 0.72, *p* < 0.01; *r* = 0.71, *p* < 0.01). In each of the significant correlations it was found that worse postural sway was associated with increased repetitive behavior and restricted interests.

## Discussion

Our work is interested in objectively characterizing the observed motor “clumsiness” in autism and how these impairments are related specifically to the core symptoms of ASD. The primary focus of this study was a systematic assessment of postural control in autism and its relationship to RRBs. RRBs can be loosely classified into lower-level (repetitive motor behaviors) and higher-level behaviors (circumscribed interests, resistance to change, rigid routines, and rituals). Our goals were to determine whether subjects with ASD had greater postural sway and whether RBS-R scores were related to the magnitude of postural sway. Poor motor control has been reported to be a predictor of repetitive behavior in individuals with mental retardation (Bodfish et al., 2001); however, the relationship between motor control and repetitive behaviors in ASD is not fully defined (Carcani-Rathwell et al., 2006).

In the current study, both the overall intensity and frequency scores on the RBS-R measure were significant predictors of COP sway areas in ASD. This was true for both lower-level and higher-level RRBs. These results are consistent with previous findings of motor impairment in ASD. Our results also support previous findings of a relationship between RRBs and postural control in individuals with mental retardation (Bodfish et al., 2001). However, we are the first to show a relationship between these behaviors and postural control in ASD.

Motor control findings in autism are compatible with the view that autism is associated with dysfunction of the motor control system mediated, at least in part, by the basal ganglia (BG), cerebellum, and associated cortico-subcortical circuitry (Dawson, 1996; Lewis and Bodfish, 1998), including the striatum and thalamus. These same regions have also been implicated in RRBs, including related cognitive functions, such as cognitive flexibility (Lopez et al., 2005). Previous imaging studies reported an association between caudate volume and repetitive behavior (Sears et al., 1999; Hollander et al., 2005; Rojas et al., 2006). Additionally, animal models indicate a synergistic role between the striatum and globus pallidus on the control of posture and repetitive circling behavior in rats (Hebb and Robertson, 1999). Previous studies by our group have demonstrated dynamic postural adjustments in children with ASD that have some similarities with findings seen in patients with Parkinson's disease (PD) (Fournier et al., 2010). Thus, findings regarding RRBs and motor abilities suggest that these behaviors appear to be controlled by, at least in part, overlapping neural systems. The findings from the current study support a model relating RRBs in autism to deficits in motor control.

While the approach in this study was simple and straightforward, findings of motor impairment in basic motor skills in children with ASD have been observed as early as infancy and within the first 2 years of life (Adrien et al., 1993; Teitelbaum et al., 1998, 2004; Baranek, 1999). This suggests that systematic observation of motor development may provide information on underlying neural development and indicate impairment, even before communicative or social deficits can be ascertained (Leary and Hill, 1996; Nayate et al., 2005). Still, it is unclear whether observed motor deficits are specific to autism. Several studies have failed to find motor differences between children with ASD and those with learning disabilities or mental retardation (Morin and Reid, 1985), general developmental delay (Provost et al., 2007), and language disorders (Noterdaeme et al., 2002). For example, three studies reported poor postural control in children with ASD (Manjiviona and Prior, 1995; Miyahara et al., 1997; Ghaziuddin and Butler, 1998), however, results from two of the studies appeared to be largely due to mental retardation, rather than specifically to autism (Minshew et al., 2004).

Reported findings of motor control abnormalities in ASD may be biased by the influence of moderating variables, such as age and IQ. Our results showed that the overall model considering group and IQ was significant; however, when considering the unique contribution of group or IQ, neither significantly predicted sway. We suspect this was for a couple of reasons. Firstly, the sample size for the study was relatively small for parceling out multiple effects. Further, we suspect that IQ and group in this study were collinear. Despite these weaknesses, there was a trend toward significance for both group and IQ effect on postural sway. The current study found a roughly bimodal distribution of postural sway area, such that half of the children with ASD performed comparable to TD controls, whereas the other half performed >2 SD outside the TD range. Our preliminary analyses found that children with impaired sway had lower IQ scores, although all had IQ scores at least within the low average range. Children with worse sway were also significantly younger, by almost 4 years. Given the younger age of the motor-impaired ASD subgroup, it would be interesting to follow these subjects longitudinally to determine whether motor impairments for some children with ASD are due to a developmental delay, whereas for others it is a developmental deviation. A longitudinal study would allow us to determine cutpoints, such that if a child with ASD continues to show basic postural impairments past a certain age, then that might indicate a developmental deviation. Regardless, future studies of motor skills in ASD should provide comparisons that control for possible moderating variables, such as age and IQ.

The specific profiles of movement abilities in ASD continue to be elucidated (Noterdaeme et al., 2002). It appears that motor control impairments may characterize a subset of individuals with ASD. Previous research suggests that the presence and severity of repetitive behaviors are likely multidetermined and serve several functions (Lewis and Kim, 2009). Because of the large heterogeneity in functioning in ASD, it will be important to conduct profile analyses to examine specific characteristics and abilities in order to continue to elucidate underlying neurobiological involvement and to guide development of treatments to address specific symptom profiles. We propose that is no longer enough to say that individuals with ASD have increased postural sway. We need to conduct in depth profile analyses of specific patterns of movement impairment within the context of several possible moderators. For example, a principal components analysis of quiet standing found that four components explained the pattern of sway in typical subjects (Rocchi et al., 2004) and a fifth component was added when examining sway in patients with PD (Rocchi et al., 2006).

In an attempt to further replicate Bodfish et al. (2001) we are gathering more data using non-linear analyses of sway to determine whether, in addition to having greater sway area, children with ASD will also show more regular, sinusoidal patterns of sway movement. Since postural stability is the basis for nearly all movements, including reaching and gait, we are beginning to examine whether children with worse sway are also more impaired on other motor abilities. Previous findings provide evidence for dysfunction in the cortical–striatal–pallidal network that controls RRBs, as well as the coordination and multisensory integration of information leading to refinements in motor functioning in response to incoming information, particularly for midline control (such as postural sway). Further these repetitive, cyclical behaviors likely co-occur because the immature motor system in ASD does not override cyclical oscillators in the CNS, which leads to protracted and enhanced expression of repetitive behaviors and poor motor control.

Postural stability is essential for the performance of nearly any motor movement. An immature postural system can be a limiting factor on the emergence of other motor skills, leading to delayed or abnormal development, which may, in turn, constrain the ability to achieve functional independence. The central nervous system must stabilize body posture before engaging in goal-directed tasks. The integrity of the postural control system becomes even more important when motor activities require dynamic modulation of the multiple joints of the body. Delayed or abnormal postural control may constrain the ability for children with autism to develop related stability or mobility skills. Research suggests that the postural system in individuals with ASD is immature and may never reach adult levels. By better characterizing impairments in postural control relative to cognitive development and RRBs, we may better design treatments that address postural instability early in development, which may help minimize or prevent subsequent emergence of deficits in other developmental abilities and perhaps the persistence of RRBs.

### Conflict of interest statement

The authors declare that the research was conducted in the absence of any commercial or financial relationships that could be construed as a potential conflict of interest.
